# The micro CT evaluation of crown and root pulp volume versus dentin thickness in teeth in postmortem interval (PMI)

**DOI:** 10.1007/s12024-024-00805-8

**Published:** 2024-03-21

**Authors:** Selcuk Cetin, Nihat Akbulut, Kaan Orhan, Burak Bilecenoglu, Mert Ocak, Emre Bayram, Ahmet Altan, Bulent Eren, Serkan Silsupur, Bedirhan Sezer Oner

**Affiliations:** 1 Faculty of Medicine, Department of Forensic Medicine, Tokat Gaziosmanpaşa University, Tokat, Turkey; 2 Faculty of Dentistry, Department of Oral and Maxillofacial Surgery, Ondokuzmayis University, Tokat, Turkey; 3 Faculty of Dentistry, Oral and Maxillofacial Radiology Department, Ankara University, Ankara, Turkey; 4 OMFS IMPATH Research Group, Department of Imaging & Pathology, Faculty of Medicine, Oral & Maxillofacial Surgery, University of Leuven, University Hospitals Leuven, Leuven, Belgium; 5 Faculty of Dentistry, Anatomy Department, Ankara University, Ankara, Turkey; 6 Faculty of Dentistry, Endodontics Department, Tokat Gaziosmanpaşa University, Tokat, Turkey; 7 Faculty of Dentistry, Oral and Maxillofacial Surgery Department, Necmettin Erbakan University, Konya, Turkey; 8 Faculty of Medicine, Department of Forensic Medicine, Kırklareli University, Kırklareli, Turkey; 9 Faculty of Dentistry, Endodontics Department, Dicle University, Diyarbakır, Turkey; 10 Faculty of Medicine, Department of Forensic Medicine, Amasya University, Amasya, Turkey

**Keywords:** Pulp volume, micro CT, Dentin thickness, Postmortem interval

## Abstract

Determining the postmortem interval (PMI) is one of the main study subjects of forensic sciences. The main purpose of this prospective in vitro study that was the Micro-CT evaluation of teeth crown and root pulp volume versus dentin thickness in terms of PMI determination. The study involved 60 female Wistar rats, with weights ranging from 270 to 320 g. These rats were grouped into six different post-mortem period categories. Following the animals’ sacrifice, they were subjected to a natural putrefaction period, with a control group, in the grounds of a sheltered garden. Hemi-mandible samples were then extracted and placed in glass tubes for Micro-CT evaluations, following the progression of putrefaction processes. The pulp volume and dentin thickness were assessed using Micro-CT, and the gathered data underwent statistical analysis. Micro-CT was employed to analyze sixty right mandibular second molar teeth in the hemi-mandible. The crown pulp volume exhibited a reduction in group 6, with a value of 0.239 mm^3^ after a three-month period of natural putrefaction (*p* < 0.001). There is statistically differences among groups in case of pairwise comparison (*p* < 0.05). However, the root pulp volume and dentin thickness variables did not display any statistically significant changes. Despite certain limitations associated with this study, the Micro-CT findings concerning teeth pulp volume can serve as an objective parameter, especially for late postmortem investigations and the estimation of time of death.

## Introduction

In the field of forensic odontology, which is a component of forensic medicine, the investigation of the postmortem interval (PMI) is a crucial aspect that researchers tackle in order to address real-world issues. This investigation is typically divided into three periods: early (0–24 h), intermediate (24 h − 7 days), and late (after 7 days). Various researchers have focused on these timeframes to find solutions [1–5]. Historically, forensic dentistry has primarily concentrated on tasks such as age estimation and postmortem toxicology [6–8]. Numerous techniques have been explored or employed within forensic dentistry to study PMI, encompassing physical examinations of external features, molecular biology, histopathological studies, thanatochemistry, entomology, spectroscopic methods, anthropology, and enzyme activity analyses utilizing diverse bodily tissues, fluids, dental hard tissues, and pulpal tissues [9–11].

The accuracy of determining the PMI is notably higher during the early period (0–24 h) compared to the intermediate and late periods, as outlined in studies [12–14]. This heightened accuracy can be attributed to the presence of more objective methods available during this phase. These methods include the assessment of rigor mortis, body cooling, and livor mortis, which can be reliably measured in the early stages of death. Additionally, the extent of tissue decomposition is considerably lesser during the early period in contrast to the late period, contributing to the improved accuracy in PMI determination [15, 16].

In the past decade, teeth have gained prominence as a valuable resource for forensic PMI investigations. This is primarily attributed to their inherent hardness and remarkable resilience against both postmortem and perimortem decomposition processes [5, 7, 10, 17]. In the existing literature, certain authors such as Alibegovic, who utilized cartilage, and Higgins and Austin, who employed DNA sourced from teeth, have recommended methods involving teeth as objective tools for determining the intermediate and late periods of PMI [18, 19]. Furthermore, teeth tissues have been employed by various researchers for estimating or determining PMI.

Micro-CT scanning has gained increasing popularity in the examination of hard tissues such as teeth and bones [20]. This technology allows for a range of analyses including measurements of mineral density, pulp volume, enamel, cement, dentin thickness, and it finds applications in various fields including tissue engineering projects [21–23].

The primary objectives of this prospective in vitro study were centered around Micro-CT evaluations of volume in teeth crowns and pulp roots, in comparison to dentin thickness, with a focus on their relevance to PMI determination or death estimation. This study aimed to establish these parameters as novel tools, particularly valuable for use in late postmortem periods.

## Materials and methods

The study protocol was approved by the Institutional Review Board and Animal Experiments Local Ethics Committee of XXXX University and the guidelines, regulations for the care and use of laboratory animals have been observed.

The study encompassed a total of 60 female Wistar rats, exhibiting weights within the range of 270 to 320 g. These rats were systematically divided into six distinct categories based on post-mortem periods, with each group consisting of 10 animals.

Following the euthanization of the animals, they were subjected to a natural putrefaction period within a sheltered garden environment, with the exception of Group 1. In order to maintain controlled weather conditions, an electric stove was utilized to regulate and monitor the temperature, maintaining an average of 25 °C. Additionally, a mercury thermometer device was employed to regularly measure the temperature intervals throughout the course of the study. The six groups were organized as follows:

Control group (Group 1): After the animals were euthanized and hemi-mandibular samples were obtained, they were immediately subjected to Micro-CT evaluations within glass tubes. In this case, the animals were not subjected to a nature putrefaction period.

Group 2: The animals in this group were allowed to undergo a nature putrefaction period of one week following their euthanization.

Group 3: The animals in this group were subjected to a nature putrefaction period of two weeks subsequent to their euthanization.

Group 4: The animals in this group were exposed to a nature putrefaction period of four weeks following their euthanization.

Group 5: The animals in this group were left to a nature putrefaction period of eight weeks after being euthanized.

Group 6: The animals in this group were subjected to a nature putrefaction period of twelve weeks after their euthanization.

In all groups except the Control group, hemi-mandible samples were obtained and subsequently placed into glass tubes for Micro-CT evaluations after undergoing the respective nature putrefaction processes.

### Micro-CT evaluation

#### Micro-CT scanning

The rat mandibles were placed into appropriately sized glass tubes, with each glass tube containing three mandibles. These samples were then transported to the Anatomy Laboratory for Micro-CT scanning and subsequent analysis. For the scanning procedure, a high-resolution desktop Micro-CT system (Bruker Skyscan 1275, Kontich, Belgium) was utilized.

#### Micro-CT imaging analysis

For visualization and quantitative measurements of the samples, the NRecon software (ver. 1.6.10.4, SkyScan, Kontich, Belgium) and CTAn (ver. 1.16.1.0, SkyScan) were employed. These software tools employed a modified algorithm based on the method described by Feldkamp et al. [24] to generate axial, two-dimensional (2D) images with a resolution of 1000 × 1000 pixels.

During the reconstruction phase, specific parameters were adjusted:

Ring artifact correction and smoothing were set to zero.

Beam artifact correction was set at 40%.

The NRecon software (SkyScan, Kontich, Belgium) was used to reconstruct the images obtained from the scanner, producing 2D slices that depicted the specimen’s internal structure. In total, 1023 cross-sectional images were reconstructed from the entire volume captured by the Micro-CT.

Furthermore, the CTAn software (SkyScan, Aartselaar, Belgium) was employed for three-dimensional (3D) volumetric visualization, analysis, and measurements of area and volume using Micro-CT data. All reconstructions were displayed on a 21.3-inch flat-panel color-active matrix TFT medical display (NEC MultiSync MD215MG, Munich, Germany) with a resolution of 2048 × 2560 pixels at 75 Hz, and a dot pitch of 0.17 mm. The display operated at 11.9 bits. After reconstruction, interpolated region of interests (ROI) using CTAn software, were drawn to include the rat 1st and 2nd molar teeth, separately. The mandible itself as well as the mandibular canals was excluded from the ROI region. Using these ROIs the crown, pulp volume and dentin thickness were measured. All specifications of the program was used in order to analyze the 2D and 3-D microarchitecture of each sample.

For each individual slice, a region of interest (ROI) was designated to exclusively encompass a single object. This enabled the calculation of thickness and volumes. All reconstructions and measurement images were executed by a dentomaxillofacial radiologist with 18 years of experience (KO).

### Statistical analysis

We used IBM SPSS Statistics for Windows, version 20.0 (IBM Corp, Armonk NY, 10,504, USA) to analyze the data we collected. We first checked the normality of all experiment groups with the Shapiro-Wilk Test. Then we performed a One Way Repeated ANOVA test to compare the six group variables. When we found significant differences between the groups, we used the Bonferroni test to do pairwise comparisons among the six groups. We set the significance level at 5% (*p* < 0.05).

## Results

Sixty right mandibular second molar teeth were extracted from the rats and subsequently subjected to Micro-CT analysis as part of this study. All data obtained throughout the study were found to exhibit homogeneity upon statistical analysis. The Shapiro-Wilk Test confirmed that the data met the criteria for homogeneity. Consequently, parametric tests were employed for the statistical analysis of all the data.

The outcomes of the statistical calculations are presented as follows:

### Crown and root pulp volume analysis

**Fig. 1:**
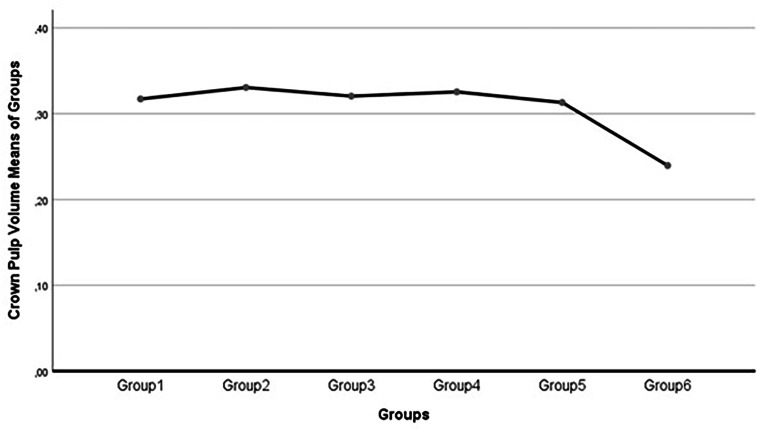
Crown pulp volume (mm^3^) course along with all groups

**Fig. 2:**
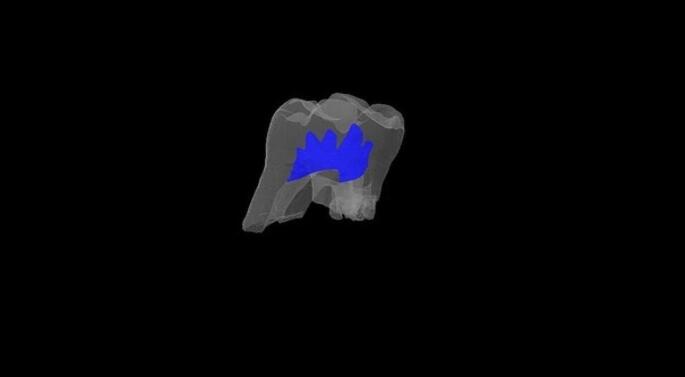
Micro-CT crown pulp volume view

In all groups, except for group 6, there were no observed changes in crown pulp volume. However, group 6 exhibited a decline in crown pulp volume, with a value of 0.239 mm3. This decrease suggests that the three-month nature putrefaction period was responsible for this outcome, although the possibility of attenuation or artifacts during Micro-CT scanning and evaluation cannot be ruled out (Tables 1 and 2; Figs. 1 and 2).

**Table 1:** The parametric Anova test used for comparison of all groups in terms of crown pulp volume variables

Measure: Group						
Source		Type III Sum of Squares	df	Mean Square	F	Sig. (*p*-value)
CrownPulpVolume	Sphericity Assumed	,058	5	,012	10,581	,000

*p* < 0.05, there is statistically differences among groups

**Table 2:** Bonferroni test used for pairwise comparison of groups

Group	Group	Sig.(*p*-value)	
1	2	1,000	
	3	1,000	
	4	1,000	
	5	1,000	
	6	,031	
2	3	1,000	
	4	1,000	
	5	1,000	
	6	,005	
3	4	1,000	
	5	1,000	
	6	,038	
4	5	1,000	
	6	,001	
5	6	,016	

*p* < 0.05, there is statistically differences among groups in case of pairwise comparison

**Fig. 3:**
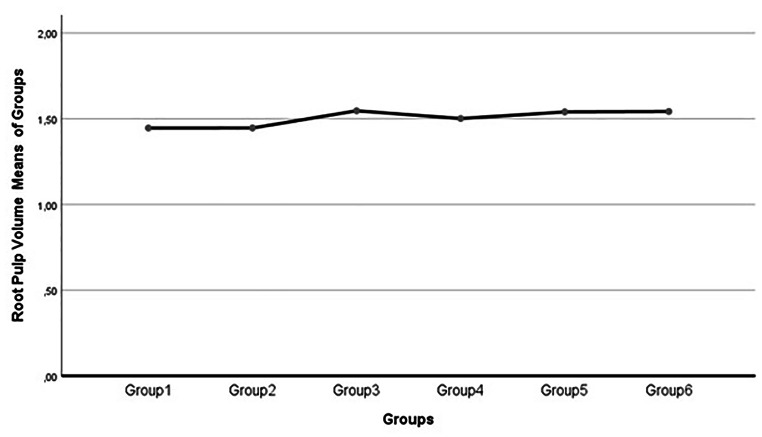
Root pulp volume values (mm^3^) course along with all groups

**Fig. 4:**
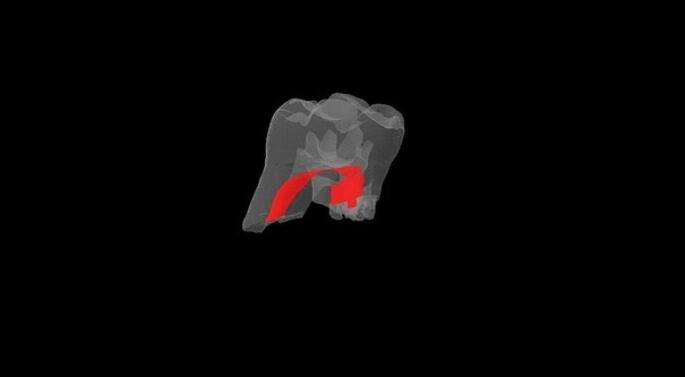
Micro-CT root pulp volume view

Statistical calculations for all groups indicated no significant alterations in root pulp volume (Table 2; Figs. 3 and 4).

### Dentin variables analysis

**Fig. 5:**
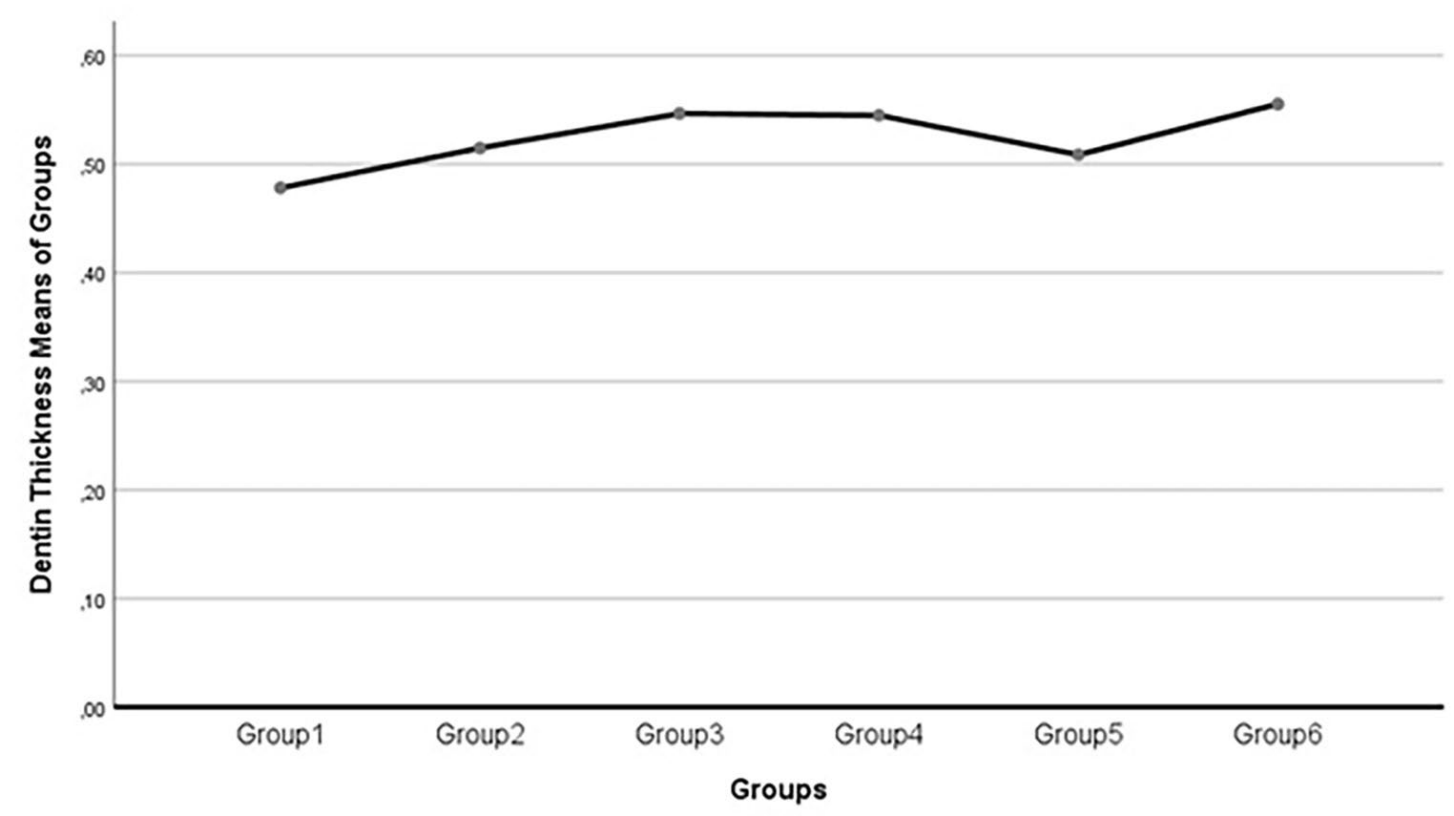
Dentin thickness values (mm) course along with all groups

**Fig. 6:**
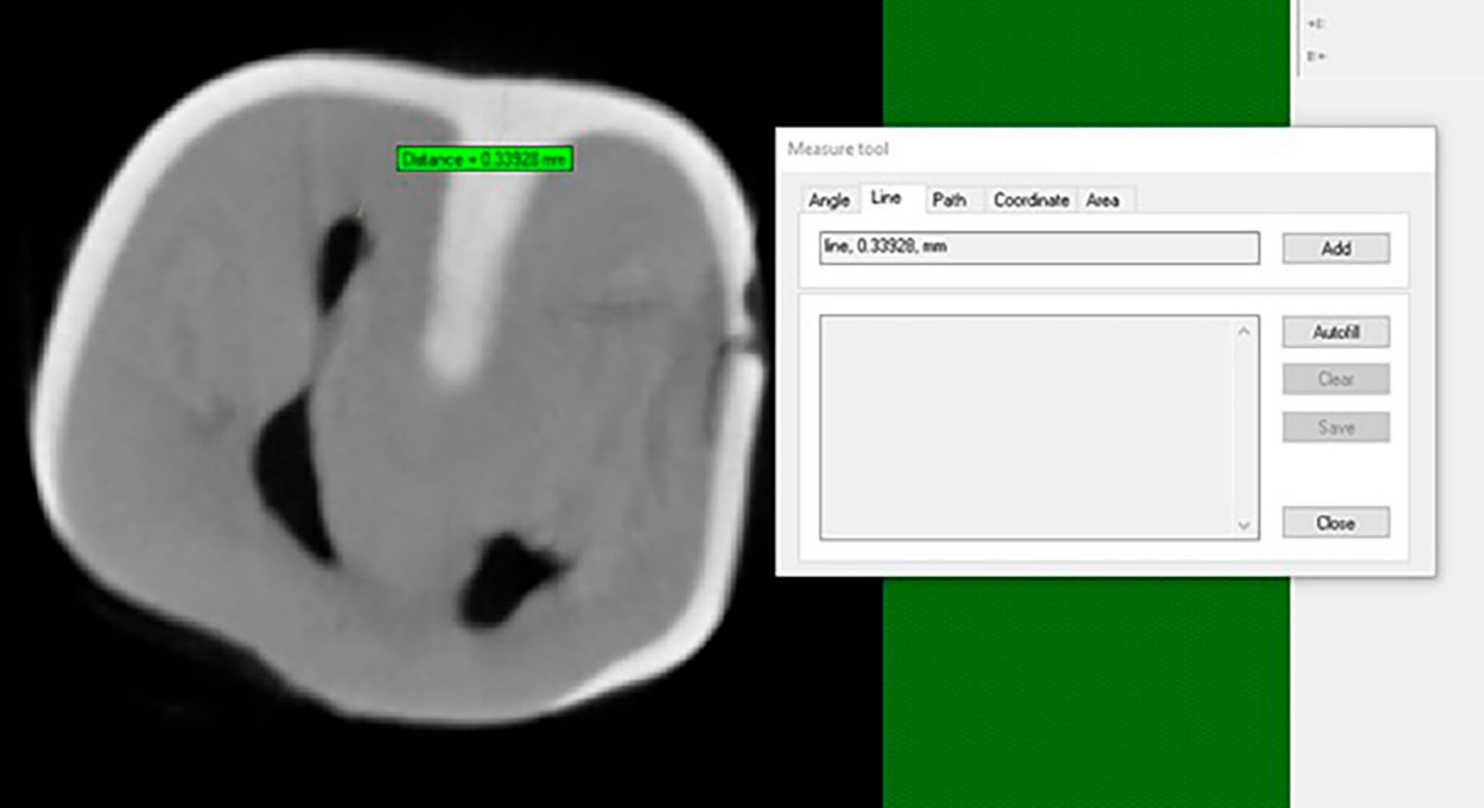
Micro-CT dentin thickness measurement view

Dentin thickness values did not exhibit statistically significant changes across all groups (Table 3; Figs. 5 and 6).

**Table 3:** The parametric Anova test used for comparison of all groups in terms of root pulp volume variables

Measure: Group						
Source		Type III Sum of Squares	df	Mean Square	F	Sig. (*p*-value)
Dentin Thickness	Sphericity Assumed	,044	5	,009	2,002	,097

*p* > 0,05; there is no difference with statically significance in terms of multivariate comparisons

## Discussion

The most notable and primary finding of this study was the decline in crown pulp volume during the final putrefaction period of group 6. In the preceding groups, the mean crown pulp volume ranged from 0.313 mm^3^ to 0.330 mm^3^, which did not exhibit statistically significant differences. However, in group 6, a distinct crown pulp volume value of 0.239 mm^3^ was observed.

The second significant finding of this study is the absence of any correlation between dentin thickness and the reduction in crown pulp volume. This suggests that changes in dentin thickness are not necessarily linked to the reduction in crown pulp volume, without any concurrent calcification processes taking place in the pulp cavity [25]. This observation is in line with our current understanding of normal physiological events in living individuals under standard conditions.

Mineralization is a complex series of events instigated by the extracellular matrix, encompassing both collagenic and non-collagenic components, matrix vesicles, intracellular mineralization, and cellular debris. These factors collectively contribute to biomineralization processes [26]. It is important to note that the terms “calcification” and “mineralization” are often used interchangeably, although they refer to distinct processes. Calcification, in contrast to mineralization, arises from physicochemical interactions between calcium and phosphate, leading to the formation of mineral phases within soft tissues. Unlike mineralization, calcification is a non-physiological condition and can manifest as the formation of mineral phases that do not resemble apatite structures. This phenomenon, which is evident after necrotic events as seen in our study samples, can result in the spontaneous collapse of non-apatite-shaped mineral complexes [27, 28].

The significant reduction in pulp volume observed during the postmortem period, such as in our group 6, could potentially be attributed to apoptotic pulp calcifications resulting from environmental conditions. Furthermore, imaging technologies like Nano, Micro, Medical, and Cone beam CT operate based on X-ray energy sources. X-ray beams traverse the subject and are ultimately detected by an X-ray detector. The X-ray energy experiences attenuation in accordance with the linear attenuation coefficient of the subject’s tissues [29]. The mentioned calcification processes may be influenced by the postmortem period, potentially leading to variations in Micro-CT results. Therefore, the observed reduction in crown pulp volume in group 6 might be influenced by X-ray attenuation errors or other factors specific to Micro-CT imaging, particularly in the crown region of teeth.

While Micro-CT investigations might exhibit discrepancies or uncertainties, particularly concerning the contradiction between dentin thickness and pulp volume, it’s worth noting that similar errors in Micro-CT investigations of dental pulp volume are likely to occur across various studies. Despite these potential limitations, the overall findings of the investigations remain consistent, and the objectivity of the results is largely unaffected.

In the field of forensic medicine and dentistry, the development of more objective methods and techniques for determining the PMI or estimating the time of death, especially during late intervals, is an ongoing effort [4, 11, 16]. In essence, within the existing literature, it’s widely recognized that obtaining objective findings during the late stages of the postmortem period can be challenging. Sun et al. [13], for instance, reported that the accuracy of PMI determination tends to decrease as the postmortem interval progresses into the late period. This underscores the need for improved and more reliable approaches for estimating PMI, particularly in the later stages after death.

Efforts to develop objective methods for determining PMI have yielded various valuable findings in the field of forensic medicine and dentistry. Alibegovic proposed that articular cartilage could serve as an objective method for determining PMI in the late postmortem period [5, 7, 14]. Yadav et al. utilized cellular changes as an objective tool in short-term postmortem gingival samples for PMI investigations [30]. Higgins et al. explored DNA rates and distributions of DNA degradation in different dental tissues during early and midterm postmortem periods [19]. Additionally, Higgins and Austin discussed the potential value of investigating postmortem variables in teeth tissues over a relevant time span for forensic medicine [19].

Aligned with the insights of Higgins and Austin, our study employed Micro-CT investigations of dental pulp and surrounding dentin, yielding valuable findings that hold potential for the future of forensic dentistry and forensic medicine. Neboda et al. highlighted the utility of Micro-CT analysis for mineral density studies in both fossilized and contemporary teeth [31]. Similarly, Durand et al. [25] endorsed the usefulness of Micro-CT in paleontological research [32]. In the context of computerized tomography (CT) studies, Sieswerda-Hoogendoorn et al. utilized CT machines to investigate the gestational age of neonaticide in PMI, highlighting the advantages of CT over autopsy in terms of outcome accuracy. They also emphasized its potential applicability in investigating late postmortem intervals, particularly due to the delayed nature of tissue decomposition in bones [33]. Just as supported by the literature mentioned, the changes in crown teeth volume through Micro-CT analysis can indeed serve as an objective parameter for PMI investigations.

Another intriguing finding of this study was the absence of changes in root pulp volume compared to crown pulp volume. The authors noted that there was limited or no existing information about this particular difference in the available literature. However, they speculated that the presence of fewer soft pulp tissues containing less collagenic components might have contributed to this outcome, potentially due to the attenuation processes inherent to Micro-CT.

## Conclusions and limitations

Considering the limitations inherent in this study, the following conclusions can be drawn: Micro-CT findings pertaining to dental pulp volume can serve as an objective parameter in future forensic investigations, particularly for cases involving late postmortem periods and death estimation. Further research is warranted to investigate the primary mechanisms underlying the decline in crown pulp volume. Employing alternative techniques in future studies could shed more light on this phenomenon. The observed differences between changes in crown pulp and root pulp volumes warrant further exploration in future dental and forensic studies. Gaining a clearer understanding of these differences could contribute to a more comprehensive comprehension of dental tissue responses in postmortem contexts.

In conclusion, the Micro-CT investigations of dental tissues conducted in this study provide preliminary findings that can offer valuable insights for guiding future directions in forensic medicine research. The observations and results from our study contribute to the ongoing development of objective tools and methods for postmortem interval determination, potentially informing the field’s advancements and enhancing its accuracy.

## Key Point

1. The Micro-CT investigations of dental tissues conducted in this study provide preliminary findings that can offer valuable insights for guiding future directions in forensic medicine research.
2. Our study contributes to the ongoing development of objective tools and methods for postmortem interval determination, potentially informing the field’s advancements and enhancing its accuracy.
3. Micro-CT findings concerning teeth pulp volume can serve as an objective parameter, especially for late postmortem investigations and the estimation of time of death.

## References

[1] Vavpotic M, Turk T, Martincic DS, Balazic J. Characteristics of the number of odontoblasts in human dental pulp post-mortem. Forensic Sci Int. 2009;193:122–6. 10.1016/j.forsciint.2009.09.023 19892501 10.1016/j.forsciint.2009.09.023

[2] Yang F, Zhang X, Hu S, et al. Changes in Microbial communities using pigs as a model for postmortem interval estimation. Microorganisms. 2023;11. 10.3390/microorganisms11112811 10.3390/microorganisms11112811 PMC10672931 38004822

[3] Bose C, Kshirsagar S, Vijayan M, et al. The role of RLIP76 in oxidative stress and mitochondrial dysfunction: evidence based on autopsy brains from Alzheimer’s disease patients. Biochim Biophys Acta Mol Basis Dis. 2024;1870:166932. 10.1016/j.bbadis.2023.166932 37926360 10.1016/j.bbadis.2023.166932

[4] Xiang QQ, Chen LF, Su Q, et al. Research Progress on Microbial Community Succession in the Postmortem interval estimation. Fa Yi Xue Za Zhi. 2023;39:399–405. 10.12116/j.issn.1004-5619.2022.420606 37859480 10.12116/j.issn.1004-5619.2022.420606

[5] Moitas B, Caldas IM, Sampaio-Maia B. Microbiology and postmortem interval: a systematic review. Forensic Sci Med Pathol. 2023. 10.1007/s12024-023-00733-z 37843744 10.1007/s12024-023-00733-z PMC11297127

[6] Roy J, Shahu U, Shirpure P, et al. A literature review on dental autopsy - an invaluable investigative technique in forensics. Autops Case Rep. 2021;11:e2021295. 10.4322/acr.2021.295 34458165 10.4322/acr.2021.295 PMC8387071

[7] Viciano J, Lopez-Lazaro S, Tanga C. Post-mortem Dental Profile as a powerful Tool in Animal Forensic Investigations-A Review. Anim (Basel). 2022;12. 10.3390/ani12162038 10.3390/ani12162038 PMC9404435 36009628

[8] Demiralp KO, Kursun Cakmak S, Aksoy S, et al. Assessment of paranasal sinus parameters according to ancient skulls’ gender and age by using cone-beam computed tomography. Folia Morphol (Warsz). 2019;78:344–50. 10.5603/FM.a2018.0089. 10.5603/FM.a2018.0089 30280374

[9] Wenzlow N, Mills D, Byrd J, Warren M, Long MT. Review of the current and potential use of biological and molecular methods for the estimation of the postmortem interval in animals and humans. J Vet Diagn Invest. 2023;35:97–108. 10.1177/10406387231153930 36744749 10.1177/10406387231153930 PMC9999395

[10] Gemmellaro MD, Hamilton GC, Ware JL. Review of Molecular Identification techniques for forensically important Diptera. J Med Entomol. 2019;56:887–902. 10.1093/jme/tjz040 31173634 10.1093/jme/tjz040

[11] Bakdash A, Kumar S, Gautam KA, Mishra VC. Use of flow cytometry in forensic medicine: current scenario and future prospects. J Forensic Leg Med. 2018;60:42–4. 10.1016/j.jflm.2018.09.010 30296631 10.1016/j.jflm.2018.09.010

[12] Woess C, Huck CW, Badzoka J, et al. Raman spectroscopy for postmortem interval estimation of human skeletal remains: a scoping review. J Biophotonics. 2023;16:e202300189. 10.1002/jbio.202300189 37494000 10.1002/jbio.202300189 PMC13631402

[13] Dawson BM, Ueland M, Carter DO, McLntyre D, Barton PS. Bridging the gap between decomposition theory and forensic research on postmortem interval. Int J Legal Med. 2023. 10.1007/s00414-023-03060-8 37491634 10.1007/s00414-023-03060-8 PMC10861637

[14] Pittner S, Ehrenfellner B, Monticelli FC, et al. Postmortem muscle protein degradation in humans as a tool for PMI delimitation. Int J Legal Med. 2016;130:1547–55. 10.1007/s00414-016-1349-9 26951243 10.1007/s00414-016-1349-9 PMC5055573

[15] Ducloyer M, David A, Dautreme B, et al. Pictorial review of the postmortem computed tomography in neonaticide cases. Int J Legal Med. 2021;135:2395–408. 10.1007/s00414-021-02677-x 34383117 10.1007/s00414-021-02677-x

[16] Ducloyer M, Tuchtan L, Delteil C, et al. Lung density measurement in postmortem computed tomography: a new tool to assess immediate neonatal breath in suspected neonaticides. Int J Legal Med. 2020;134:1159–66. 10.1007/s00414-019-02103-3 31286205 10.1007/s00414-019-02103-3

[17] Srirangarajan S, Sindhu V, Raju S, et al. Evaluation of gingival tissue samples for predicting the time of death using histological and biochemical tests. Forensic Sci Int. 2021;324:110850. 10.1016/j.forsciint.2021.110850 34082395 10.1016/j.forsciint.2021.110850

[18] Alibegovic A. Cartilage: a new parameter for the determination of the postmortem interval? J Forensic Leg Med. 2014;27:39–45. 10.1016/j.jflm.2014.08.005 25287798 10.1016/j.jflm.2014.08.005

[19] Higgins D, Austin JJ. Teeth as a source of DNA for forensic identification of human remains: a review. Sci Justice. 2013;53:433–41. 10.1016/j.scijus.2013.06.001 24188345 10.1016/j.scijus.2013.06.001

[20] Baier-Stegmaier S, Gundlach C, Chriel M, et al. Computed tomography as a Method for Age determination of Carnivora and odontocetes with Validation from individuals with known age. Anim (Basel). 2023;13. 10.3390/ani13111783 10.3390/ani13111783 PMC10251996 37889740

[21] Sevgi U, Johnsen GF, Hussain B, et al. Morphometric micro-CT study of contralateral mandibular incisors. Clin Oral Investig. 2023;28:20. 10.1007/s00784-023-05419-y 38147175 10.1007/s00784-023-05419-y PMC10751267

[22] Monteiro JL, Takusagawa T, Sampaio GC, et al. Gelatin methacryloyl hydrogel with and without dental pulp stem cells for TMJ regeneration: an in vivo study in rabbits. J Oral Rehabil. 2024;51:394–403. 10.1111/joor.13608 37830126 10.1111/joor.13608

[23] Qiao X, Tang J, Dou L, et al. Dental Pulp Stem Cell-Derived exosomes regulate anti-inflammatory and Osteogenesis in Periodontal Ligament Stem cells and promote the repair of experimental periodontitis in rats. Int J Nanomed. 2023;18:4683–703. 10.2147/IJN.S420967 10.2147/IJN.S420967 PMC10441659 37608819

[24] Feldkamp LA, Goldstein SA, Parfitt AM, Jesion G, Kleerekoper M. The direct examination of three-dimensional bone architecture in vitro by computed tomography. J Bone Min Res. 1989;4:3–11. 10.1002/jbmr.5650040103 10.1002/jbmr.5650040103 2718776

[25] Jannati R, Afshari M, Moosazadeh M, et al. Prevalence of pulp stones: a systematic review and meta-analysis. J Evid Based Med. 2019;12:133–9. 10.1111/jebm.12331 30461204 10.1111/jebm.12331

[26] Uskokovic V. When 1 + 1 > 2: Nanostructured composites for hard tissue engineering applications. Mater Sci Eng C Mater Biol Appl. 2015;57:434–51. 10.1016/j.msec.2015.07.050 26354283 10.1016/j.msec.2015.07.050 PMC4567690

[27] Kadulkar N, Kataki R, Deka A, et al. Comparative evaluation of the Effect of different Chelating agents on Mineral Content and Erosion of Radicular Dentine: a FESEM-EDS analysis. Eur Endod J. 2024;9:73–80. 10.14744/eej.2023.19971 38157281 10.14744/eej.2023.19971 PMC10777092

[28] Cruz-Maya I, Altobelli R, Alvarez-Perez MA, Guarino V. Mineralized microgels via Electrohydrodynamic atomization: optimization and in Vitro Model for dentin-pulp complex. Gels. 2023;9. 10.3390/gels9110846 10.3390/gels9110846 PMC10670945 37998935

[29] Ashton JR, West JL, Badea CT. In vivo small animal micro-CT using nanoparticle contrast agents. Front Pharmacol. 2015;6:256. 10.3389/fphar.2015.00256 26581654 10.3389/fphar.2015.00256 PMC4631946

[30] Yadav AB, Angadi PV, Kale AD, Yadav SK. Histological assessment of cellular changes in postmortem gingival specimens for estimation of time since death. J Forensic Odontostomatol. 2015;33:19–26. 26851446 PMC5734814

[31] Neboda C, Anthonappa RP, King NM. Tooth mineral density of different types of hypomineralised molars: a micro-CT analysis. Eur Arch Paediatr Dent. 2017;18:377–83. 10.1007/s40368-017-0306-8 29081019 10.1007/s40368-017-0306-8

[32] Durand S, Dufour J, Rosas A, Becce F, Orr C. Three-dimensional comparative study of human bipartite scaphoids and the Os Centrale of the wrist in Neandertals and Non-human Anthropoid Primates. Diagnostics (Basel). 2021;11. 10.3390/diagnostics11122295 10.3390/diagnostics11122295 PMC8700597 34943532

[33] Sieswerda-Hoogendoorn T, Soerdjbalie-Maikoe V, Maes A, van Rijn RR. The value of post-mortem CT in neonaticide in case of severe decomposition: description of 12 cases. Forensic Sci Int. 2013;233:298–303. 10.1016/j.forsciint.2013.09.023 24314533 10.1016/j.forsciint.2013.09.023

